# A randomized, 29-day, dose-ranging, efficacy and safety study of daprodustat, administered three times weekly in patients with anemia on hemodialysis

**DOI:** 10.1186/s12882-019-1547-z

**Published:** 2019-10-16

**Authors:** Christine K. Bailey, Stephen Caltabiano, Alexander R. Cobitz, Chun Huang, Kelly M. Mahar, Vickas V. Patel

**Affiliations:** 1 R&D, Clinical Sciences, GlaxoSmithKline, 1250 S. Collegeville Road, Mail Code UP 4200, Collegeville, PA 19426 USA; 2 R&D, GlaxoSmithKline, 1250 S. Collegeville Road, Collegeville, PA 19426 USA; 3 R&D, Clinical Statistics, GlaxoSmithKline, 1250 S. Collegeville Road, Collegeville, PA 19426 USA; 4 R&D, Clinical Pharmacology, Modeling and Simulation, GlaxoSmithKline, 1250 S. Collegeville Road, Collegeville, PA 19426 USA; 5 R&D, Discovery Medicine, GlaxoSmithKline, 1250 S. Collegeville Road, Collegeville, PA 19426 USA

**Keywords:** Anemia, Hemodialysis, Three-times weekly (TIW), Dose response, Bayesian, Efficacy, Clinical trials, Safety

## Abstract

**Background:**

Daprodustat is a hypoxia-inducible factor-prolyl hydroxylase inhibitor currently being investigated as a treatment for anemia of chronic kidney disease (CKD) in both dialysis and nondialysis patients. In clinical studies to date, daprodustat has been administered orally as a once-daily regimen. This randomized, double-blind, placebo-controlled study characterized the initial dose-hemoglobin response as well as the efficacy and safety of three times weekly (TIW) daprodustat in hemodialysis patients switched from stable recombinant human erythropoietin (rhEPO), in accordance with a TIW hemodialysis schedule.

**Methods:**

103 patients on hemodialysis with baseline hemoglobin of 9.0 to 11.5 g/dL and previously receiving a stable dose of rhEPO or its analogs were randomized 1:1:1:1:1 to receive daprodustat 10, 15, 25, or 30 mg or placebo TIW over 29 days.

**Results:**

Mean baseline hemoglobin was 10.6 g/dL for the placebo group and each daprodustat cohort. Daprodustat produced dose-dependent changes in mean hemoglobin from baseline to day 29. Using a Bayesian approach, the estimated dose conversion ratio between once-daily and TIW daprodustat was ~ 2.0 across the evaluated dose range using an E_max_ model. Daprodustat was generally well tolerated, with an adverse event (AE) profile consistent with the hemodialysis population.

**Conclusions:**

These data help inform the appropriate dose conversion ratio to be applied to daily doses to obtain equivalent daprodustat TIW doses and suggest TIW treatment with daprodustat can treat anemia of CKD safely, supporting future long-term studies for this indication using a TIW dosing regimen.

**Trial registration:**

ClinicalTrials.gov Identifier: NCT02689206; date registered: 02/11/2016.

**Electronic supplementary material:**

The online version of this article (10.1186/s12882-019-1547-z) contains supplementary material, which is available to authorized users.

## Background

The causes of anemia in patients with chronic kidney disease (CKD) are multi-factorial, including relative or absolute deficiency of erythropoietin (EPO), reduced iron availability related to chronic inflammation, and gastrointestinal blood loss [1, 2]. Anemia is further exacerbated by shortened erythrocyte survival, which is associated with the uremic milieu and the hemodialysis procedure [1]. While effective therapies are available for anemia of CKD, including supplemental iron, recombinant human EPO (rhEPO) and its analogs, and blood transfusions, each has significant limitations [3–5]. Specifically, adverse cardiovascular events have been reported with rhEPO and its analogs, which led the US Food and Drug Administration to limit use of these treatments in patients on hemodialysis with hemoglobin levels < 10 g/dL, and the dose should be reduced or the drug discontinued when hemoglobin exceeds 11 g/dL [3].

Hypoxia-inducible factor (HIF)-prolyl hydroxylase inhibitors (PHIs) are an emerging new class of therapy for the treatment of anemia of CKD. These molecules stimulate erythropoiesis through inhibition of HIF-prolyl hydroxylase domain enzymes (PHD1, PHD2, PHD3) resulting in the accumulation of HIFα transcription factors and increased expression of HIF-responsive genes under normoxic conditions [6]. HIF-responsive genes include EPO and other proteins involved in increasing oxygen availability and utilization as well as proteins involved in iron utilization, angiogenesis, apoptosis, metabolism, and vascular tone [7]. Based on their mechanism of action, HIF-PHIs are postulated to be associated with fewer major adverse cardiovascular events by raising hemoglobin without the supraphysiologic EPO concentrations associated with rhEPO therapy [6].

Daprodustat is a HIF-PHI currently being investigated as a treatment for anemia of CKD in both dialysis and non-dialysis patients [6, 8]. In clinical studies to date, daprodustat has been administered orally as a once-daily regimen [8, 9]. However, the preferred practice in countries that utilize a three times weekly (TIW) hemodialysis schedule is to administer anemia therapy at the time of the hemodialysis session [10].

The rationale of this study was to assess the feasibility of administering daprodustat in conjunction with a TIW hemodialysis schedule, and determine the equivalent TIW doses of daprodustat required to maintain hemoglobin levels in patients previously stable and responding to therapy with rhEPO or its analogs who are switched to daprodustat. The primary objective was to characterize the initial hemoglobin dose-response relationship for TIW dosing of daprodustat at day 29 and to describe the relationship between once-daily and TIW dosing of daprodustat in hemodialysis-dependent patients with anemia of CKD who are switched from a stable dose of rhEPO or its analogs. Additional objectives included estimation of the dose conversion ratio between once-daily and TIW daprodustat; characterization of the pharmacodynamic (PD) effect of daprodustat TIW dose regimens on EPO, vascular endothelial growth factor (VEGF), markers of iron metabolism, and indices of hematopoiesis; and assessment of the pharmacokinetics (PK), safety, and tolerability of daprodustat TIW.

## Methods

### Study design

This was a randomized, double-blinded, dose-ranging, placebo-controlled, parallel group study. The study consisted of a 28-day screening period, a 29-day treatment period (with study visits on days 1, 15, and 29), and a follow-up visit approximately 14 days after completing treatment. For patients on dialysis TIW, the day 1, 15, and 29 study visits could not occur on the first dialysis session following the longest intradialytic interval.

The dose-response relationship for once-daily dosing of daprodustat in the hemodialysis population was established in a 24-week phase 2 study (ClinicalTrials.gov identifier NCT01977482) [9]. In that study, the hemoglobin response (change from baseline) was determined at 4 weeks over the dose range of 4 mg to 12 mg; therefore, in order to make a direct comparison between the once-daily and TIW regimens, a 29-day treatment period was utilized.

The study was initiated on February 17, 2016 and completed on January 25, 2017. Twenty-nine centers in 5 countries randomized at least one participant: 8 centers in the Russian Federation, 7 each in Spain and the United States, 4 in Canada, and 3 in Germany.

Study participants were stratified by low or high prior dose of rhEPO (or its analogs) based on the average weekly dose during the 12 weeks prior to randomization (day 1). Low dose was defined as < 100 IU/kg/week epoetin or < 0.5 μg/kg/week darbepoetin. High dose was defined as ≥100 IU/kg/week epoetin or ≥ 0.5 μg/kg/week darbepoetin. Participants were randomized in equal proportions to a daprodustat dose or placebo within each stratum without using prior rhEPO dose to determine the daprodustat dose.

Participants discontinued their current rhEPO therapy prior to day 1 and were randomized by a computer-generated randomization schedule using the Prism randomization system in a 1:1:1:1:1 ratio to receive oral daprodustat 10, 15, 25, or 30 mg or placebo TIW on a dialysis day. Evidence with epoetin alfa (Epogen), has shown that increases in dosing interval can be achieved by a proportionate increase in the dose administered at each interval [11]. As the daprodustat dose response with hemoglobin was generally linear between 4 and 24 mg once daily, it was anticipated that a similar time-proportional efficacy relationship would exist for daprodustat and the management of hemoglobin levels would remain as the dosing was extended from once every day to three times weekly. Based on the hypothesis that the total weekly TIW dose that achieves the same total weekly once-daily dose would have the same Hgb effect, the TIW dose would be equivalent to ~ 2.3 times the once-daily dose (7 doses/ week × 1 week/ 3 doses = 2.3 multiplier), doses of 10, 15, 25, and 30 mg TIW were investigated for 29 days to most closely match the 4, 6, 10, and 12 mg once-daily doses that were evaluated in the study by Meadowcroft et al. [9]. Studying multiple doses allowed a robust analysis of a range of TIW doses to determine the “equivalent” ratio to bridge to once-daily dose levels. The dose-hemoglobin response relationship was generally linear in the once-daily dose range of interest (4 to 24 mg) but it was not known if this was maintained with the higher doses needed for TIW dosing.

Each participant was assigned a randomization number by the interactive voice and web response system, which was managed by Pharmaceutical Product Development, LLC, Wilmington, NC, USA. Once a randomization number was assigned, it could not be reassigned. Study participants, investigators, and site and sponsor staff were all blinded to the treatment assignment. To maintain the blind, each participant took one tablet of study treatment from each of three bottles of study treatment, on each dosing day. The combination of tablets provided the appropriate dose of study treatment, and included a combination of tablet strengths, active and placebo, to correspond to the appropriate dose. An internal Safety Review Team (SRT), which included GlaxoSmithKline personnel involved in the conduct of the study, monitored blinded safety data in-stream during the study.

### Study population

ndividuals who were at least 18 years old and had stable baseline hemoglobin values between 9.0 g/dL and 11.5 g/dL based on average values obtained on day − 28, day − 14, and day 1 were eligible for the study. Study participants were also required to be on a stable hemodialysis regimen of 3 to 5 times weekly for at least 90 days, with a single-pool Kt/V_urea_ of ≥1.2. Additional eligibility criteria included that participants be on a stable dose of rhEPO or its analogs, with the total weekly dose varying by no more than 50%, during the 4 weeks prior to day − 28. A protocol amendment on May 11, 2016 updated the eligibility criteria to include the complete required timeframe for participants to be on a stable dose of rhEPO or its analogs up to 4 weeks prior to day − 28 through day 1. Those participants who were receiving maintenance oral or intravenous (IV) iron supplementation were also required to be on a stable dose from 4 weeks prior to day − 28 through day 29.

Patients were ineligible for the study if they were being treated with ≥360 IU/kg/week IV or ≥ 250 IU/kg/week subcutaneous (SC) epoetin, ≥1.8 μg/kg/week IV or SC darbepoetin, or ≥ 2.2 μg/kg/week methoxy polyethylene glycol-epoetin beta within the 8 weeks prior to randomization. Patients with low values of vitamin B12 (< 200 pg/mL [148 pmol/L]), folate (< 2.0 ng/mL [4.5 nmol/L]), ferritin (< 100 ng/mL [225 pmol/L]), or transferrin saturation (TSAT) (< 20%) were also ineligible. Additional exclusion criteria included a planned change in dialysis modality or renal transplant within the study time period; significant cardiovascular, hematologic, hepatic, or chronic inflammatory disease; active gastrointestinal (GI) bleeding or GI bleeding within 8 weeks prior to day − 28 through day 1; QT interval corrected for heart rate using Bazett’s formula (QTcB) > 500 msec or QTcB > 530 msec in patients with bundle branch block; and malignancy within 2 years of screening, currently receiving treatment for cancer, or a known complex kidney cyst (ie, Bosniak Category II F, III, or IV) > 3 cm.

### Withdrawal criteria

Study medication was permanently discontinued if participants met the hemoglobin stopping criteria, based on HemoCue measured hemoglobin values as follows: a confirmed hemoglobin < 7.5 g/dL, a confirmed hemoglobin increase ≥1.0 g/dL over the previous 2 weeks, or a confirmed hemoglobin decrease ≥2.0 g/dL over the previous 2 weeks based on point of care hemoglobin measurements. For confirmed hemoglobin ≥13.0 g/dL, a decision regarding withdrawal was made in consultation with the GlaxoSmithKline Medical Monitor. Central lab hemoglobin values were also analyzed retrospectively to identify how many participants met each of the hemoglobin stopping criteria.

Participants were also permanently discontinued from study treatment if they received a blood transfusion or kidney transplant, became or intended to become pregnant, had active GI bleeding, were diagnosed with cancer (with the exception of localized squamous cell and basal cell carcinoma), or missed 2 consecutive dialysis sessions. Liver chemistry was also monitored during the study.

Participants who discontinued study treatment completed an early withdrawal assessment and follow-up visit 14 ± 3 days after the last dose of study treatment.

### Study analyses

No formal sample size or power calculations were performed. The focus of this study was on characterizing the hemoglobin-TIW dose-response relationship and using the results to estimate the dose conversion ratio between once-daily and TIW doses that would produce the same hemoglobin response, not on formal hypothesis testing. A sample size of approximately 90 randomized participants was determined based on feasibility (18 per treatment group).

The primary study endpoint was change in hemoglobin from baseline at day 29. Baseline hemoglobin was defined as the average of the hemoglobin measured at the day − 14 and day 1 visits. This change from the planned analysis, which defined baseline hemoglobin as the day 1 hemoglobin value, was made in order to reflect the overall pretreatment stable hemoglobin value and was prespecified in the Reporting and Analysis Plan, which was finalized before database freeze and unblinding.

The dose-response relationship between daprodustat TIW and hemoglobin at day 29 was characterized using a three-parameter E_max_ model fitted using Bayesian statistical methods. E_max_ was assumed to be an appropriate model based on the biology of daprodustat and once-daily dose-response modeling completed to date for daprodustat. Prior distributions for each of the model parameters (E0, E_max_, ED50, and variance) used in this trial were informed by the once-daily dose-response from a previous phase 2 study (ClinicalTrials.gov identifier NCT01977482) [9] This modeling provided confidence in setting prior distributions for E0, however, there was greater uncertainty in the estimation of E_max_ and ED50, which was explored in sensitivity analyses with alternative (weaker) prior distributions, including a Bayesian three-parameter E_max_ model based on the completer population, Bayesian three-parameter E_max_ model using sensitivity (weaker, less informative) priors, and linear dose-response model based on the intention-to-treat (ITT) population.

Once the dose-response model was fitted satisfactorily, the posterior distribution of the model parameters (E0, E_max_, ED50, and variance) was summarized through means, standard deviation (SD), median, Monte Carlo standard error (MCSE), MCSE/SD and equal-sided 95% credibility intervals. The predicted value of hemoglobin change from baseline at day 29 at a series of doses (range of possible doses from 0 to 50 was sectioned off into a sequence of 0.5 increments; ie, dose levels of 0, 0.5, 1.0, 1.5, 2.0….50) was also monitored and the median and 95% credibility values obtained at each dose.

The observed change in hemoglobin from baseline at day 29 was used as the response variable in the E_max_ model. Participants who had a day 15 hemoglobin measurement, but a missing day 29 hemoglobin measurement, were included with a day 29 value imputed using 2 × (day 15 hemoglobin – baseline hemoglobin). Participants who prematurely discontinued dosing prior to day 15 were nonevaluable for the dose-response analysis.

The dose conversion ratio of the once-daily dose to the corresponding TIW dose that produced an equivalent hemoglobin response was estimated based on the dose-hemoglobin response relationship for the once-daily regimen derived from a previous study [9] and for the TIW regimen derived from the current trial. TIW dosing and once-daily dosing were both estimated by Bayesian E_max_ model.

The observed change in hemoglobin from baseline to day 29 was analyzed by a repeated measures model using the PROC MIXED procedure. Model effects included baseline hemoglobin, treatment, time, and treatment-by-time interactions. The variance-covariance structures for repeated measures within the same subject were unstructured. For day 29, least squares mean differences were calculated as estimates of the difference between the daprodustat cohorts and placebo, together with 95% confidence intervals (CIs).

Additional secondary endpoints included observed change from baseline in hepcidin, ferritin, transferrin, total iron, unsaturated iron binding capacity (UIBC), total iron binding capacity (TIBC), hematocrit, red blood cell (RBC) count, reticulocyte count, and reticulocyte hemoglobin (CHr), which were summarized by visit (day 1, 15, and 29) and treatment group. Additionally, maximum observed change from baseline in plasma EPO and maximum observed percent change from baseline in VEGF were assessed and summarized by treatment group. Descriptive PK summaries of daprodustat and major metabolites were also summarized by daprodustat dose level. In general, secondary analyses were descriptive and estimation based.

Safety assessments included incidence and severity of AEs and serious AEs (SAEs), reasons for discontinuation of investigational product, discontinuations for safety-related reasons, and absolute values and changes from baseline over time in laboratory parameters, electrocardiograms (ECGs), and vital signs. Dose-dependent AE relationship was not assessed in this study. The investigator or site staff was responsible for detecting, documenting, and reporting AEs/SAEs. AEs were collected from the start of study treatment (day 1) and until the follow-up visit. SAEs were recorded from the time of subject consent to participate in the study up to and including any follow-up contact.

## Results

### Patient population

**Fig. 1:**
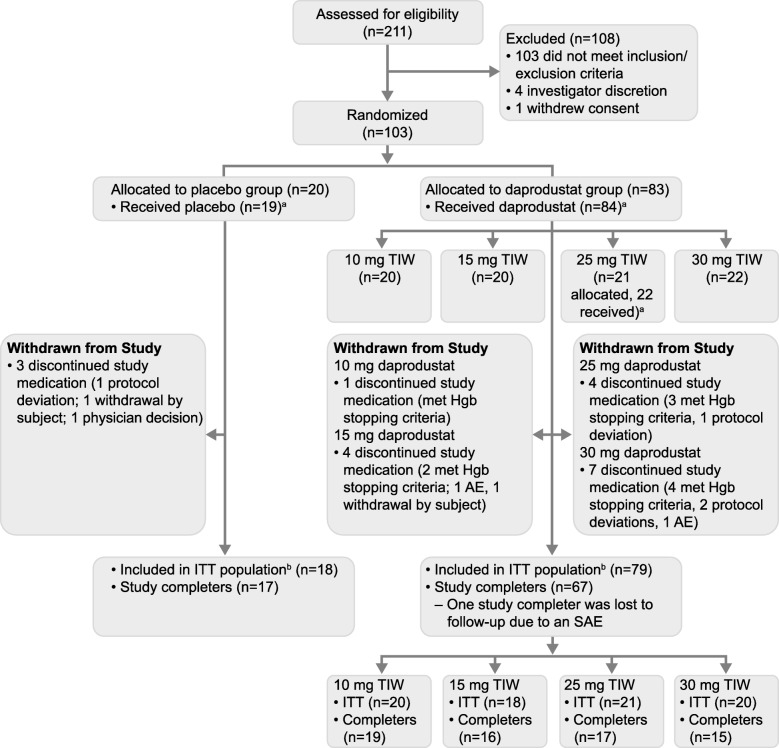
Study Flow Diagram. AE, adverse event; ITT, intention-to-treat; Hgb, hemoglobin; PK, pharmacokinetics; SAE, serious adverse event; TIW, three times weekly. ^a^One participant randomized to the placebo group erroneously received daprodustat 25 mg. This participant was counted in the placebo group in the ITT population and in the daprodustat 25 mg cohort in the safety and PK populations. ^b^The ITT population comprised all randomized participants who received at least one dose of study treatment, and had a baseline and at least one corresponding on-treatment assessment including hemoglobin

Of 211 patients screened, 103 (49%) met the eligibility criteria and were randomized to the placebo group (*n* = 20) or one of the four daprodustat cohorts (*n* = 83 in total) (Fig. 1). All 103 patients were included in the safety and PK populations and 97 were included in the ITT population (18 in the placebo group and 79 in the total daprodustat group). One participant randomized to the placebo group erroneously received daprodustat 25 mg. This participant was counted in the placebo group in the ITT population and in the daprodustat 25 mg cohort in the safety and PK populations. Of all patients randomized, 17 participants in the placebo group (85%) and 67 in the total daprodustat group (81%) completed the treatment period. With the exception of 1 participant in the total daprodustat group, all patients who completed the study also completed the follow-up visit.

The most common reason for study treatment discontinuation in the total daprodustat group was due to participants reaching protocol-defined hemoglobin stopping criteria. No participant receiving placebo discontinued study treatment due to meeting protocol-defined hemoglobin stopping criteria.

Demographic and baseline characteristics were generally balanced between the placebo and total daprodustat group, with the exception that the total daprodustat group contained more males (Table 1). The majority of participants in both the placebo and total daprodustat group were white, the mean baseline hemoglobin level was 10.6 g/dL, and approximately half of the participants in both groups had diabetes.

**Table 1:** Demographic and Baseline Characteristics (ITT Population)

	Placebo *n* = 18	Total Daprodustat *n* = 79	Daprodustat Dose			
			10 mg *n* = 20	15 mg *n* = 18	25 mg *n* = 21	30 mg *n* = 20
Age (years)						
Mean ± SD	60.9 ± 13.3	64.1 ± 15.0	63.6 ± 17.0	59.1 ± 12.7	67.2 ± 15.2	65.9 ± 14.3
Sex, *n* (%)						
Male	7 (39)	50 (63)	12 (60)	13 (72)	12 (57)	13 (65)
Race, *n* (%)						
White	12 (67)	53 (67)	12 (60)	11 (61)	15 (71)	15 (75)
African American	6 (33)	21 (27)	6 (30)	6 (33)	5 (24)	4 (20)
Asian	0	2 (3)	1 (5)	1 (6)	0	0
Other	0	3 (4)	1 (5)	0	1 (5)	1 (5)
Weight (kg)						
Mean ± SD	82.8 ± 26.5	78.8 ± 22.5	79.8 ± 26.0	79.8 ± 22.3	79.1 ± 24.3	76.3 ± 18.1
BMI (kg/m^2^)						
Mean ± SD	30.6 ± 8.6	28.2 ± 7.5	28.7 ± 8.6	27.4 ± 7.8	29.4 ± 7.4	27.1 ± 6.4
Diabetes, *n* (%)						
Yes	9 (50)	35 (44)	6 (30)	8 (44)	9 (43)	12 (60)
Hgb (g/dL)						
Mean ± SD	10.6 ± 0.8	10.6 ± 0.7	10.6 ± 0.7	10.7 ± 0.8	10.4 ± 0.6	10.5 ± 0.7
Stratification factor, *n* (%)						
Low prior rhEPO dose^a^	14 (78)	59 (75)	15 (75)	14 (78)	16 (76)	14 (70)
High prior rhEPO dose^b^	4 (22)	20 (25)	5 (25)	4 (22)	5 (24)	6 (30)
Type of rhEPO, *n* (%)						
Darbepoetin	1 (6)	20 (25)	4 (20)	3 (17)	7 (33)	6 (30)
Epoetin	17 (94)	59 (75)	16 (80)	15 (83)	14 (67)	14 (70)
Standardized prior rhEPO dose (IU/kg/week)						
Mean ± SD	66.8 ± 43.1	86.6 ± 88.4	108.0 ± 147.1	75.0 ± 41.3	69.4 ± 51.2	93.7 ± 72.0
Smoking history,^c^ *n* (%)	*N* = 19	*N* = 84	*N* = 20	*N* = 20	*N* = 22	*N* = 22
Never	15 (79)	54 (64)	14 (70)	13 (65)	15 (68)	12 (55)
Current	1 (5)	4 (5)	2 (10)	1 (5)	0	1 (5)
Former	3 (16)	26 (31)	4 (20)	6 (30)	7 (32)	9 (41)
Mode of dialysis,^c^ *n* (%)	*N* = 19	*N* = 84	*N* = 20	*N* = 20	*N* = 22	*N* = 22
Hemodiafiltration	3 (16)	18 (21)	3 (15)	3 (15)	6 (27)	6 (27)
Hemodialysis	16 (84)	66 (79)	17 (85)	17 (85)	16 (73)	16 (73)

*BMI* body mass index, *Hgb* hemoglobin, *rhEPO* recombinant human erythropoietin, *ITT* intention-to-treat, *SD* standard deviation

^a^Low dose: < 100 IU/kg/week epoetin or < 0.5 μg/kg/week darbepoetin or < 0.6 μg/kg/week methoxy polyethylene glycol-epoetin beta

^b^High dose: ≥100 IU/kg/week epoetin or ≥ 0.5 μg/kg/week darbepoetin or ≥ 0.6 μg/kg/week methoxy polyethylene glycol-epoetin beta

^c^Safety population

### Change in hemoglobin from baseline to day 29 and over time

**Fig. 2:**
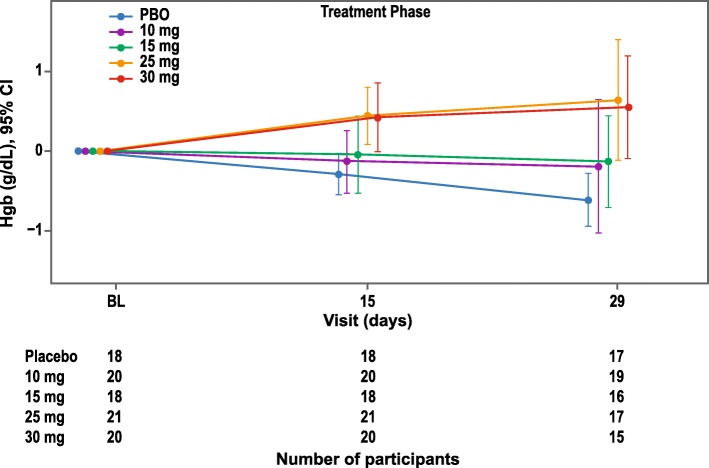
Mean Change from Baseline and 95% CI for Hemoglobin (g/dL) Over Time (ITT Population). BL, baseline; CI, confidence interval; Hgb, hemoglobin; ITT, intention-to-treat

Switching from rhEPO to daprodustat produced dose-dependent mean changes in hemoglobin (g/dL) from baseline to day 29 (Fig. 2 and Additional file 1: Table S1). Mean hemoglobin increased over time in the daprodustat 25 mg and 30 mg cohorts, while mean hemoglobin remained near baseline levels in the 10 mg and 15 mg cohorts, and decreased in the placebo group. The model-adjusted treatment difference from placebo demonstrated that the treatment effect on hemoglobin generally increased with the dose levels of daprodustat (Additional file 1: Table S2).

### Dose-response model and dose conversion

**Fig. 3:**
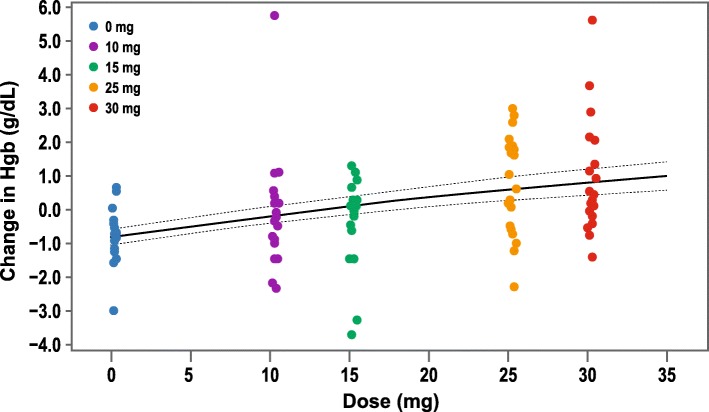
Posterior E_max_ Dose-Response Curve for Hemoglobin (g/dL) Change from Baseline at Day 29 (ITT Population). The two dotted lines are 95% credible bands. E_max_, maximal response; Hgb, hemoglobin; ITT, intention-to-treat

The dose-response relationship was well characterized within the dose range evaluated in this study (0–30 mg daprodustat administered TIW). There was high variability in individual participant’s hemoglobin response within treatment groups, and a large overlap in hemoglobin response between adjacent doses (Fig. 3). Overall, mean hemoglobin response increased with increasing daprodustat dose.

The dose-response relationship for once-daily dosing of daprodustat has been previously described [9]. The dose conversion ratio between once-daily dosing and TIW dosing of daprodustat was estimated to be ~ 2.0 across the evaluated dose range when the estimation was based on the Bayesian three-parameter E_max_ model (Table 2).

**Table 2:** Summary of Dose Ratio Between Three-Times Weekly Doses and Once-Daily Doses and Estimated Doses Corresponding to Specific Hemoglobin Response Levels (ITT Population)

Estimated TIW Doses (mg)	95% CI	Selected Hemoglobin Response	Estimated QD Doses (mg)	95% CI	Dose Ratio (TIW/QD)^a^	95% CI (Bootstrap)
2.77	0.00–5.82	a change of -0.5 g/dL (minimally effective dose^b^)	1.27	0.00–2.63	2.19	0.49–5.20
6.44	3.21–9.99	a change of -0.25 g/dL	3.16	1.58–4.42	2.04	0.95–4.54
10.48	6.98–15.01	a change of 0 g/dL (target dose^c^)	5.26	3.91–6.62	1.99	1.25–3.17
14.93	10.71–20.97	a change of 0.25 g/dL	7.58	6.18–9.45	1.97	1.33–2.92
19.85	14.59–27.96	a change of 0.5 g/dL	10.18	8.40–13.08	1.95	1.32–2.89
25.30	18.74–36.16	a change of 0.75 g/dL	13.09	10.68–17.60	1.93	1.27–2.92
31.38	23.27–45.95	a change of 1 g/dL	16.38	13.08–23.33	1.92	1.21–2.98

*CI* confidence interval, *ITT* intention-to-treat, *QD* once daily; *TIW*, three times weekly

^a^Both the TIW doses and the QD doses were estimated by Bayesian E_max_ model

^b^The smallest dose that achieves a change of −0.5 g/dL in Hgb over 29 days after being switched from ESA

^c^The dose that achieves a 0 g/dL change in Hgb over 29 days after being switched from rhEPO or its analogs

The target dose (dose expected to cause a 0 g/dL change in hemoglobin) estimated by the three-parameter Bayesian E_max_ model was 10.48 mg daprodustat administered TIW (Table 2).

### Participants who reached the hemoglobin stopping criteria

Ten participants were withdrawn from the study based on HemoCue measured hemoglobin values during the study. In a retrospective analysis of central laboratory hemoglobin values, 2 participants experienced decreases that met protocol-defined hemoglobin stopping criteria (1 participant in 10 mg cohort and 1 in 15 mg cohort, but none in placebo). Overall, 17 participants (1 placebo participant who mistakenly received 25 mg daprodustat and 16 daprodustat participants) experienced increases that met the protocol-defined hemoglobin stopping criteria. Most of these 17 participants had received either 25 mg or 30 mg daprodustat (14 participants), with the remaining 3 participants having been in the two lower daprodustat dose cohorts. The majority (11/13) of participants with a ≥ 1 g/dL increase in hemoglobin over the previous 2 weeks, including 9 of 11 participants who received either 25 mg or 30 mg daprodustat, were in the low prior rhEPO (and its analogs) dose stratum.

### EPO and VEGF

**Fig. 4:**
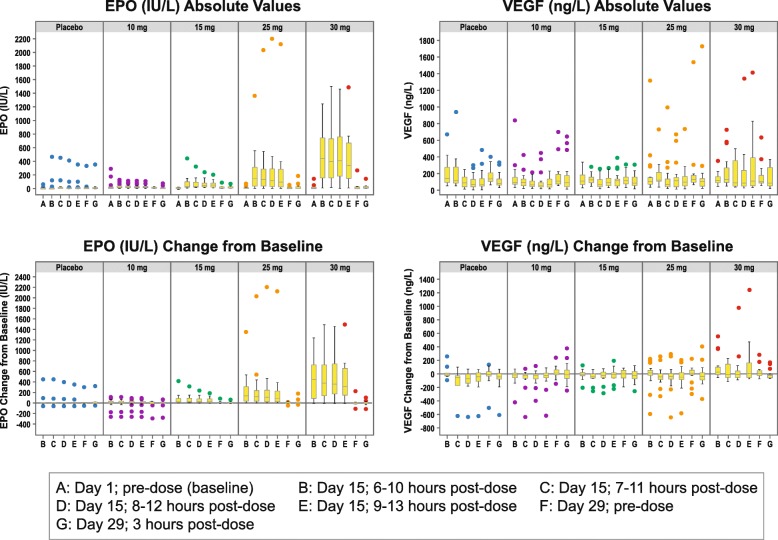
EPO and VEGF Absolute Values and Change From Baseline by Visit and Sample Time (ITT Population; Post-Hoc Analyses). Each boxplot displays the distribution of the parameter indicated on the Y-axis label. The bottom and top edges of the box indicate the intra-quartile range (IQR); that is the range of values between the 25th and 75th percentiles. The line inside the box indicates the median value. The whiskers are drawn from the box to the most extreme point that is less than or equal to 1.5 times the IQR. The dots are outliers that are more extreme than the upper and lower fences (±1.5 IQR). EPO, erythropoietin; ITT, intention-to-treat; VEGF, vascular endothelial growth factor

Median baseline concentrations of plasma EPO were similar across treatment groups. There was a dose-dependent increase in the observed circulating EPO concentration following administration of daprodustat TIW (Fig. 4). Day 29 pre-dose EPO levels were near or below baseline values in participants receiving daprodustat, indicating no accumulation of EPO after daprodustat TIW treatment.

Median baseline plasma VEGF concentrations were similar among the daprodustat cohorts, but somewhat higher in the placebo group (Fig. 4). Circulating VEGF concentrations were highly variable in all dose cohorts at all time points. Day 29 pre-dose VEGF concentrations in participants receiving daprodustat were near or below baseline, indicating no accumulation of VEGF after administration of daprodustat TIW.

### Markers of Iron metabolism and utilization

Both geometric mean baseline hepcidin and mean baseline ferritin values were similar across treatment groups and each daprodustat dose cohort. Daprodustat administration led to dose-dependent decreases from baseline at day 29 in geometric mean hepcidin and in mean ferritin (Additional file 1: Figure S1).

Mean baseline values for transferrin, TIBC, UIBC, and CHr were also similar across all treatment groups and trended toward increases in a dose-dependent manner during the study (Additional file 1: Table S3). Both mean baseline total iron and mean baseline TSAT were similar across treatment groups, and there were overall no clinically relevant changes from baseline for either parameter.

### Indices of hematopoiesis

Mean baseline hematocrit and RBC counts were similar across all treatment groups. Daprodustat administration led to a dose-dependent mean change from baseline in these parameters (Additional file 1: Table S4). Participants in the placebo group had a mean decrease in hematocrit and RBCs. Mean baseline reticulocyte counts were also similar across treatment groups and daprodustat trended toward dose-dependent mean changes from baseline. For the daprodustat 25 mg and 30 mg cohorts, the observed increase was smaller at day 29 than at day 15.

### Pharmacokinetic results

Daprodustat was observed to be readily absorbed with a median time to maximum observed plasma concentration (t_max_) value of approximately 2.0 h (Additional file 1: Table S5). Systemic exposures (area under the concentration-time curve from time 0 to time t [AUC_0-t_]) increased with dose, and were approximately linear with dose across the dose range (ie, for a 3-fold increase in dose from 10 mg to 30 mg, AUC increased 3.2-fold). Geometric mean maximum observed plasma concentration (C_max_) exposures were similar between the 10 mg and 15 mg doses and higher for the 25 mg and 30 mg doses. High inter-participant variability was noted for each PK parameter, with calculated % coefficient of variation (CV) values > 90%.

### Safety analysis

Overall, 26% of participants in the placebo group and 33% in the combined daprodustat group experienced at least one on-treatment AE (Table 3). In both groups, the majority of on-treatment AEs were mild or moderate in intensity. There was no dose-dependent relationship between AEs and daprodustat. The most frequently reported on-treatment AE in the total daprodustat group was hypotension (3 participants, 4%). Three on-treatment AEs assessed by the investigator to be related to daprodustat were reported in 2 participants: dizziness and gastroesophageal reflux disease in 1 participant in the daprodustat 10 mg cohort, and depression in 1 participant in the daprodustat 15 mg cohort. No treatment-related AEs were reported in the placebo group.

**Table 3:** Adverse Events (Safety Population)

Preferred Term, *n* (%)	Placebo *n* = 19	Total Daprodustat *n* = 84	Daprodustat Dose			
			10 mg *n* = 20	15 mg *n* = 20	25 mg *n* = 22	30 mg *n* = 22
On-Treatment Common AEs ≥ 2 Participants^a^						
Any Event	**5 (26)**	**28 (33)**	**9 (45)**	**6 (30)**	**7 (32)**	**6 (27)**
Hypotension	0	3 (4)	1 (5)	1 (5)	1 (5)	0
Angina unstable	0	2 (2)	1 (5)	0	0	1 (5)
Cardiac failure congestive	0	2 (2)	2 (10)	0	0	0
Diarrhea	0	2 (2)	2 (10)	0	0	0
Fluid overload	0	2 (2)	0	0	1 (5)	1 (5)
Procedural hypotension	0	2 (2)	1 (5)	0	1 (5)	0
On-Treatment Serious AEs^b^						
Any Event	**2 (11)**	**8 (10)**	**3 (15)**	**2 (10)**	**1 (5)**	**2 (9)**
Angina unstable	0	2 (2)	1 (5)	0	0	1 (5)^c^
Cardiac failure congestive	0	2 (2)	2 (10)^d^	0	0	0
Fluid overload	0	2 (2)	0	0	1 (5)^d^	1 (5)
Hypertensive crisis	0	1 (1)	0	1 (5)^c^	0	0
Mesenteric artery stenosis	0	1 (1)	0	1 (5)	0	0
Postprocedural myocardial infarction	0	1 (1)	1 (5)	0	0	0
Arteriovenous fistula thrombosis	1 (5)	0	0	0	0	0
Orthostatic hypotension	1 (5)^d^	0	0	0	0	0

*AE* adverse event

^a^Common AE is defined as an AE with ≥2 participants in any treatment group or ≥ 2% (based on unrounded value) in the total daprodustat group

^b^Participants could have ≥1 AE

^c^Study-withdrawn or drug-withdrawn

^d^Drug-interrupted

There was a similar proportion of on-treatment SAEs reported in the total daprodustat group (10%, 8 participants) and placebo group (11%, 2 participants) (Table 3). Three SAEs in the daprodustat group occurred in more than 1 participant (unstable angina, congestive cardiac failure, and fluid overload). All events resolved within 1 week. None of the SAEs were considered by the investigator to be related to study treatment.

Two participants were withdrawn from study treatment due to an SAE; both participants had been randomized to one of the daprodustat arms. One participant in the 15 mg cohort experienced a hypertensive crisis and 1 participant in the 30 mg cohort experienced unstable angina. Both events resolved in 2 days. Neither event was considered by the investigator to be related to study treatment.

No adverse trends were apparent following review of clinical laboratory values, vital sign values, and ECG values, and no deaths were reported in the randomized population.

## Discussion

This was a dose-ranging study evaluating TIW dosing of daprodustat in patients with anemia of CKD on hemodialysis in order to estimate the dose conversion ratio between once-daily and TIW administration of daprodustat and help inform TIW doses for future studies. The enrolled study population was representative of the real-world hemodialysis population, which includes ~ 30% African Americans. The results showed a dose-dependent increase in hemoglobin with TIW daprodustat, and demonstrated the ability of TIW daprodustat doses between 10 mg and 30 mg to initially maintain hemoglobin levels in patients switched from rhEPO or its analogs. The dose conversion ratio for once-daily to TIW daprodustat was estimated to be approximately 2.0 across the evaluated dose range. These data can be used to calculate the TIW doses for future phase 3 studies evaluating long-term safety and efficacy of daprodustat TIW in patients with CKD, which would allow for greater dosing flexibility and dosing aligned with a TIW hemodialysis schedule.

Since this is the first clinical study examining the effects of TIW daprodustat in the target patient population, it was important to determine the effects these higher doses of daprodustat may have on relevant PD markers. The study results are consistent with the mechanism of action of daprodustat to stabilize HIF, and similar to what has been observed with daprodustat once daily, including dose-dependent changes in plasma EPO levels, over the 29-day treatment period without subsequent accumulation even at doses that increased hemoglobin levels. In contrast, due to high variability, there was no consistent dose-dependent relationship observed in plasma VEGF levels. Changes observed in markers of iron metabolism and indices of erythropoiesis were also consistent with what would be expected with increased erythropoiesis, and were similar to the trends seen with daprodustat once daily.

A higher proportion of participants treated with daprodustat permanently discontinued study treatment due to reaching protocol-defined hemoglobin stopping criteria. However, the majority of those participants with hemoglobin increases were receiving lower levels of rhEPO or its analogs prior to entry into the study. Therefore, it is not unexpected that in this randomized dose-ranging trial, treatment with doses of daprodustat higher than required to maintain hemoglobin resulted in hemoglobin elevations requiring stopping dosing. This is less likely to occur when the prior EPO dose is used to determine the starting dose for daprodustat TIW on an individual basis.

Daprodustat TIW was generally well tolerated and raised no new safety concerns at doses up to 30 mg. Furthermore, the AEs observed are those typically seen in hemodialysis-dependent patients and occurred with a similar frequency in the placebo group and daprodustat treatment group.

Limitations of this study are the small sample size and limited treatment duration which are inherent of a dose-ranging trial. Additional limitations include the use of imputed values in the analysis for several participants who met the hemoglobin stopping criteria. Additionally, although the baseline characteristics were reflective of the hemodialysis population, there were more males in the total daprodustat group than in the placebo group.

## Conclusions

This study estimated the dose conversion ratio for once-daily to TIW daprodustat to be ~ 2.0 across the evaluated dose range. The study also demonstrated the efficacy and safety of daprodustat TIW. A dose-dependent increase in hemoglobin levels was seen when daprodustat was administered TIW. The hemoglobin effects were achieved without accumulation of plasma EPO or VEGF and with a safety profile consistent with the hemodialysis patient population. These data support future longer-term clinical studies in patients on hemodialysis to further explore daprodustat TIW to treat anemia of CKD.

## Additional files

Additional file 1: **Table S1.** Hemoglobin Over Time and Change from Baseline (ITT Population). **Table S2.** Repeated Measure Analysis for Hemoglobin Change from Baseline at Day 29 (ITT Population). **Figure S1.** A) Geometric Mean Change from Baseline and 95% CI for Hepcidin (ITT Population) and B) Mean Change from Baseline and 95% CI for Ferritin (ITT Population). **Table S3.** Change from Baseline in Markers of Iron Metabolism at Day 29 (ITT Population). **Table S4.** Change from Baseline in Indices of Hematopoiesis (ITT Population)**. Table S5.** Summary of Plasma Daprodustat Pharmacokinetic Parameters Following Dose Administration of Daprodustat (PK Population). (PDF 339 kb)

Additional file 2: Participating research centers and IEC/IRB committees. (PDF 94 kb)

## Data Availability

Portions of the dataset(s) generated or analyzed during this study are included in this published article and its supplemental information file. The datasets generated and/or analyzed during the current study will be available within 6 months of this publication, anonymized individual participant data, the annotated case report form, protocol, reporting and analysis plan, data set specifications, raw dataset, analysis-ready dataset, and clinical study report will be available for research proposals approved by an independent review committee. Proposals should be submitted to www.clinicalstudydatarequest.com. A data access agreement will be required.

## Abbreviations

AE: Adverse event
AUC_0-**∞**_: Area under the concentration-time curve from time 0 to time infinity
AUC_0-t_: Area under the concentration-time curve from time 0 to time t
BL: Baseline
BMI: Body mass index
CHr: Reticulocyte hemoglobin
CI: Confidence interval
CKD: Chronic kidney disease
C_max_: Maximum observed plasma concentration
CV: Coefficient of variation
ECG: Electrocardiogram
EPO: Erythropoietin
GI: Gastrointestinal
Hgb: Hemoglobin
HIF: Hypoxia-inducible factor
ITT: Intention-to-treat
IV: Intravenous
MCSE: Monte Carlo standard error
PBO: Placebo
PD: Pharmacodynamic
PHI: Prolyl hydroxylase inhibitor
PK: Pharmacokinetic
QD: Once daily
QTcB: QT interval corrected for heart rate using Bazett’s formula
RBC: Red blood cell
rhEPO: recombinant human erythropoietin
SAE: Serious adverse event
SC: Subcutaneous
SD: Standard deviation
SRT: Safety Review Team
t_1/2_: half-life
TIBC: Total iron binding capacity
TIW: Three times weekly
t_max_: time to maximum observed plasma concentration
TSAT: Transferrin saturation
UIBC: Unsaturated iron binding capacity
VEGF: Vascular endothelial growth factor

## References

[1] Babitt JL, Lin HY (2012). Mechanisms of anemia in CKD. J Am Soc Nephrol.

[2] Zumbrennen-Bullough K, Babitt JL (2014). The iron cycle in chronic kidney disease (CKD): from genetics and experimental models to CKD patients. Nephrol Dial Transplant.

[3] FDA Drug Safety Communication: Modified dosing recommendations to improve the safe use of erythropoiesis-stimulating agents (ESAs) in chronic kidney disease. Safety announcement issued June 24, 2011. http://www.fda.gov/Drugs/DrugSafety/ucm259639.htm#sa. Accessed 12 Mar 2019.

[4] Kidney disease: improving global outcomes (KDIGO) anemia work group (2012). KDIGO clinical practice guideline for anemia in chronic kidney disease. Kidney Int Suppl.

[5] Agarwal R, Kusek JW, Pappas MK (2015). A randomized trial of intravenous and oral iron in chronic kidney disease. Kidney Int.

[6] Gupta N, Wish JB (2017). Hypoxia-inducible factor prolyl hydroxylase inhibitors: a potential new treatment for anemia in patients with CKD. Am J Kidney Dis.

[7] Haase VH (2013). Mechanisms of hypoxia responses in renal tissue. J Am Soc Nephrol.

[8] Holdstock L, Meadowcroft AM, Maier R, Johnson BM, Jones D, Rastogi A (2016). Four-week studies of oral hypoxia-inducible factor-prolyl hydroxylase inhibitor GSK1278863 for treatment of anemia. J Am Soc Nephrol.

[9] Meadowcroft AM, Cizman B, Holdstock L, Biswas N, Johnson BM, Jones D (2019). Daprodustat for anemia: a 24-week, open-label, randomized controlled trial in participants on hemodialysis. Clin Kidney J.

[10] Dialysis Outcomes and Practice Patterns Study Program: DOPPS Practice Monitor. August 2015. http://www.dopps.org/DPM. Accessed 12 Mar 2019.

[11] McGowan T, Vaccaro NM, Beaver JS, Massarella J, Wolfson M (2008). Pharmacokinetic and pharmacodynamic profiles of extended dosing of epoetin alfa in anemic patients who have chronic kidney disease and are not on dialysis. Clin J Am Soc Nephrol.

