# Supplementary material for: A randomized, 29-day, dose-ranging, efficacy and safety study of daprodustat, administered three times weekly in patients with anemia on hemodialysis
Source: BMC Nephrol. 2019 Oct 16;20:372. doi: 10.1186/s12882-019-1547-z (PMC6796426; doi:10.1186/s12882-019-1547-z)
Supplement: Supplementary file 1 — Table S1. Hemoglobin Over Time and Change from Baseline (ITT Population). Table S2. Repeated Measure Analysis for Hemoglobin Change from Baseline at Day 29 (ITT Population). Figure S1. A) Geometric Mean Change from Baseline and 95% CI for Hepcidin (ITT Population) and B) Mean Change from Baseline and 95% CI for Ferritin (ITT Population). Table S3. Change from Baseline in Markers of Iron Metabolism at Day 29 (ITT Population). Table S4. Change from Baseline in Indices of Hematopoiesis (ITT Population). Table S5. Summary of Plasma Daprodustat Pharmacokinetic Parameters Following Dose Administration of Daprodustat (PK Population). (PDF 339 kb) [file 12882_2019_1547_MOESM1_ESM.pdf]

Additional file 1

Table S1. Hemoglobin Over Time and Change from Baseline (ITT Population)

Hgb (g/dL)	Placebo	Daprodustat Total	Daprodustat Dose			
			10 mg	15 mg	25 mg	30 mg
**Baseline,[a] n**	18	79	20	18	21	20
Mean±SD	10.6±0.75	10.6±0.69	10.6±0.71	10.7±0.77	10.4±0.59	10.5±0.73
**Day 15, n**	18	79	20	18	21	20
Mean±SD	10.3±0.97	10.7±0.93	10.5±1.19	10.6±0.87	10.9±0.78	10.9±0.85
Change from Baseline						
Mean±SD	−0.3±0.52	0.2±0.91	−0.1±0.84	0.0±0.97	0.5±0.79	0.4±0.93
**Day 29, n**	17	67	19	16	17	15
Mean±SD	10.0±1.12	10.8±1.40	10.4±1.83	10.5±0.93	11.1±1.33	11.3±1.15
Change from Baseline						
Mean±SD	−0.6±0.65	0.2±1.43	−0.2±1.75	−0.1±1.09	0.6±1.45	0.6±1.17
**Day 29 (imputed),[b] n**	18	79	20	18	21	20
Mean±SD	9.8±1.34	11.0±1.54	10.5±1.85	10.4±1.22	11.3±1.36	11.7±1.39
Change from Baseline						
Mean±SD	−0.7±0.83	0.5±1.68	−0.1±1.73	−0.2±1.36	0.9±1.47	1.2±1.74

Hgb, hemoglobin; *ITT*, intention-to-treat; *SD*, standard deviation.

[a]The average of hemoglobin measured by the central lab at week −2 and day 1 visits.

[b]Including participants (1 on placebo and 12 on daprodustat) who had a day 15 hemoglobin value but were missing a day 29 value.

Table S2. Repeated Measure Analysis for Hemoglobin Change from Baseline at Day 29 (ITT Population)

		Daprodustat Dose			
**Hgb (g/dL)**	**Placebo**	**10 mg**	**15 mg**	**25 mg**	**30 mg**
Model-Adjusted Change from Baseline at Day 29[a]					
Adjusted mean±SE (95% CI)	−0.7±0.32 (−1.33 to −0.06)	−0.1±0.30 (−0.73–0.48)	−0.2±0.32 (−0.83–0.46)	0.8±0.31 (0.16–1.38)	1.0±0.32 (0.33–1.61)
Model-Adjusted Difference from Placebo[a]					
Adjusted mean (95% CI)	--	0.6 (−0.31–1.45)	0.5 (−0.40–1.42)	1.5 (0.59–2.35)	1.7 (0.77–2.57)

CI, confidence interval; *Hgb*, hemoglobin; *ITT,* intention-to-treat; *SE*, standard error.

[a]Using a repeated measures model with terms included for baseline, treatment, time, and treatment*time. Least squares mean estimates and treatment differences of daprodustat group to placebo along with 95% CI are provided.

Figure S1. A) Geometric Mean Change from Baseline and 95% CI for Hepcidin (ITT Population) and B) Mean Change from Baseline and 95% CI for Ferritin (ITT Population)

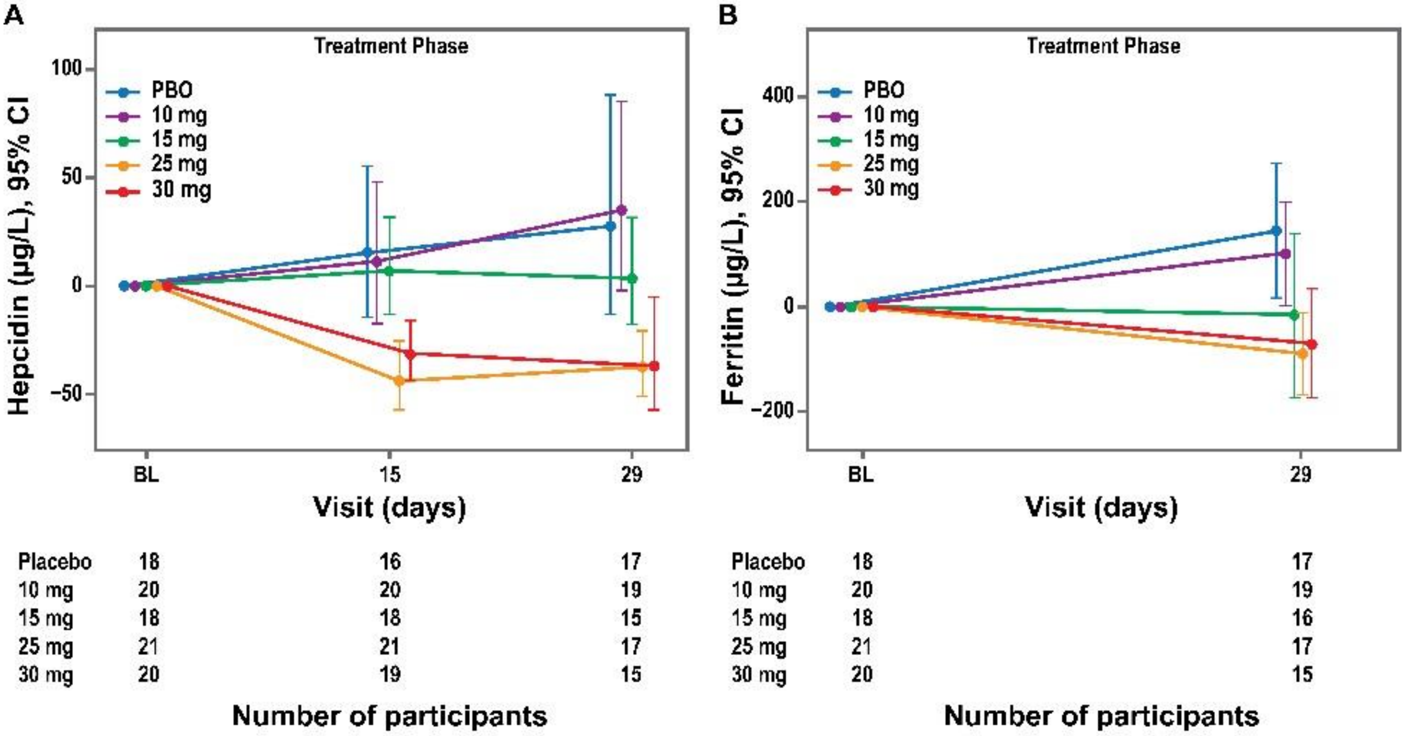

BL, baseline; *CI*, confidence interval; *ITT*, intention-to-treat; *PBO*, placebo.

Table S3. Change from Baseline in Markers of Iron Metabolism at Day 29 (ITT Population)

	Placebo	Daprodustat Total	Daprodustat Dose			
			10 mg	15 mg	25 mg	30 mg
**Transferrin (g/L)**						
**Baseline[a], n**	**18**	**79**	**20**	**18**	**21**	**20**
Mean±SD	1.6±0.36	1.6±0.30	1.6±0.28	1.6±0.39	1.6±0.29	1.6±0.26
**Day 29, n**	**17**	**67**	**19**	**16**	**17**	**15**
Mean±SD	1.7±0.33	1.7±0.40	1.6±0.25	1.9± 0.44	1.7±0.40	1.9±0.44
Absolute Change from Baseline						
Mean±SD	0.06±0.265	0.12±0.313	−0.01±0.238	0.15±0.246	0.08±0.379	0.31±0.309
**TIBC (μmol/L)**						
**Baseline[a], n**	**18**	**79**	**20**	**18**	**21**	**20**
Mean±SD	39.8±7.97	37.7±6.47	36.7±6.51	38.1±8.66	37.9±5.29	38.1±5.60
**Day 29, n**	**17**	**67**	**19**	**16**	**17**	**15**
Mean±SD	39.2±7.00	41.3±7.44	36.9±5.85	43.8±8.54	41.1±6.55	44.6±6.73
Absolute Change from Baseline						
Mean±SD	0.1±3.42	3.7±4.52	0.5±4.72	4.3±3.11	3.5±3.36	7.2±4.13
**UIBC (μmol/L)**						
**Baseline[a], n**	**18**	**79**	**20**	**18**	**21**	**20**
Mean±SD	25.9±6.50	25.5±6.21	25.3±6.05	25.8±7.04	26.0±6.09	25.1±6.13
**Day 29, n**	**17**	**67**	**19**	**16**	**17**	**15**
Mean±SD	23.6±7.58	27.5±8.20	24.6±6.84	27.8±10.91	29.1±7.30	28.9±7.13
Absolute Change from Baseline						
Mean±SD	−1.9±6.67	2.1±7.51	−0.4±7.38	1.1±8.56	3.6±5.04	4.6±8.28
**CHr (pg)**						
**Baseline[a], n**	**18**	**79**	**20**	**18**	**21**	**20**
Mean±SD	32.1±2.19	31.4±1.86	31.6±1.84	31.1±2.06	31.2±1.57	31.5±2.05
**Day 29, n**	**16**	**64**	**19**	**14**	**16**	**15**
Mean±SD	31.6±2.03	31.7±1.89	32.0±1.41	31.4±1.95	31.5±2.04	32.0±2.27
Absolute Change from Baseline						
Mean±SD	–0.2±1.38	0.3±1.22	0.2±1.34	0.3±1.10	0.2±1.04	0.6±1.44
**Total Iron (μmol/L)**						
**Baseline[a], n**	**18**	**79**	**20**	**18**	**21**	**20**
Mean±SD	13.9±4.38	12.1±4.62	11.4±4.32	12.3±4.35	11.9±5.33	13.0±4.54
**Day 29, n**	**17**	**67**	**19**	**16**	**17**	**15**

Mean±SD	15.6±6.85	13.9±6.19	12.4±5.59	16.0±6.44	11.9±4.53	15.7±7.51
Absolute Change from Baseline						
Mean±SD	2.0±6.08	1.6±6.97	0.9±6.72	3.2±8.11	−0.1±5.85	2.6±7.27
**TSAT (%)**						
**Baseline[a], n**	**18**	**79**	**20**	**18**	**21**	**20**
Geometric mean (CV%)	33.7 (26.95)	30.7 (35.66)	28.9 (42.76)	31.4 (29.03)	29.9 (38.66)	32.7 (31.61)
**Day 29, n**	**17**	**67**	**19**	**16**	**17**	**15**
Geometric mean (CV%)	36.9 (42.82)	30.6 (49.05)	30.0 (54.01)	34.5 (45.38)	26.9 (46.69)	31.8 (49.99)
Relative % Change from Baseline						
geometric mean	9.9	−1.7	2.7	9.7	−12.9	−4.9

CHr, reticulocyte hemoglobin; *CV*, coefficient of variation; *ITT*, intention-to-treat; *SD*, standard deviation; *TIBC*, total iron binding capacity; *TSAT*, transferrin saturation; *UIBC*, unsaturated iron binding capacity.

[a]The day 1 pre-dose value.

Table S4. Change from Baseline in Indices of Hematopoiesis (ITT Population)

	Placebo	Daprodustat Total	Daprodustat Dose			
			10 mg	15 mg	25 mg	30 mg
**Hematocrit (%)**						
**Baseline[a], n**	**18**	**79**	**20**	**18**	**21**	**20**
Mean±SD	33.0±3.39	32.8±3.02	33.1±2.31	33.7±4.31	32.5±2.78	32.1±2.43
**Day 15, n**	**17**	**77**	**19**	**18**	**20**	**20**
Mean±SD	32.4±2.88	33.3±3.25	32.4±3.71	32.6±2.82	34.2±3.09	33.9±3.15
Absolute Change from Baseline						
Mean±SD	−1.0±2.00	0.5±3.63	−0.7±2.19	−1.2±4.94	1.7±2.75	1.8±3.38
**Day 29, n**	**16**	**64**	**19**	**14**	**16**	**15**
Mean±SD	31.3±3.61	33.2±4.62	31.7±6.19	32.4±2.97	34.1±4.00	34.9±3.73
Absolute Change from Baseline						
Mean±SD	−2.2±2.47	0.2±5.01	−1.3±5.40	−1.5±5.38	1.5±4.51	2.2±3.85
**RBC Count (10^{12}/L)**						
**Baseline[a], n**	**18**	**79**	**20**	**18**	**21**	**20**
Mean±SD	3.4±0.37	3.5±0.38	3.5±0.30	3.6±0.49	3.4±0.31	3.4±0.38
**Day 15, n**	**17**	**76**	**19**	**17**	**20**	**20**
Mean±SD	3.4±0.31	3.5±0.38	3.4±0.41	3.5±0.34	3.5±0.29	3.5±0.47
Absolute Change from Baseline						
Mean±SD	−0.1±0.18	0.0±0.32	−0.1±0.21	−0.1±0.46	0.1±0.25	0.10±0.30
**Day 29, n**	**16**	**64**	**19**	**14**	**16**	**15**
Mean±SD	3.3±0.43	3.5±0.51	3.3±0.57	3.5±0.46	3.6±0.40	3.6±0.55
Absolute Change from Baseline						
Mean±SD	−0.2±0.26	0.0±0.45	−0.1±0.49	−0.1±0.50	0.1±0.42	0.2±0.34
**Reticulocyte Count (%)**						
**Baseline[a], n**	**18**	**79**	**20**	**18**	**21**	**20**
Mean±SD	1.6±0.57	1.7±0.68	1.7±0.53	1.6±0.70	1.6±0.65	1.8±0.86
**Day 15, n**	**17**	**76**	**20**	**16**	**20**	**20**
Mean±SD	1.2±0.53	2.0±1.14	1.4±0.73	1.8±0.82	2.5±1.46	2.4±1.03
Absolute Change from Baseline						
Mean±SD	−0.4±0.94	0.3±1.18	–0.3±0.83	0.1±0.56	0.9±1.56	0.7±1.12
**Day 29, n**	**16**	**63**	**18**	**14**	**16**	**15**
Mean±SD	1.5±0.62	1.8±0.82	1.6±0.78	1.7±0.98	1.9±0.75	1.9±0.81
Absolute Change from Baseline						
Mean±SD	−0.1±0.85	0.1±0.69	−0.1±0.80	0.0±0.58	0.4±0.55	0.1±0.70

ITT, intention-to-treat; *RBC*, red blood cells; *SD*, standard deviation.

[a]The day 1 pre-dose value.

Table S5. Summary of Plasma Daprodustat Pharmacokinetic Parameters Following Dose Administration of Daprodustat (PK Population)

Daprodustat Dose	PK Parameter[a]					
	n	C_{max} (ng/mL)	$AUC_{(0-t)}$ (ng.h/mL)	$AUC_{(0-\infty)}$ (ng.h/mL)	t_{max}[b] (h)	$t_{1/2}$ (h)
10 mg	19	140 (104.0)	312 (91.4)	348 (78.2)[c]	2.9 (1.0–12.5)	2.1 (50.0)[c]
15 mg	15	141 (248.8)	417 (168.6)	384 (142.7)[d]	2.0 (0.8–8.3)	1.9 (62.3)[d]
25 mg	16	247 (311.5)	514 (396.1)	1214 (41.6)[e]	2.0 (0.9–10.1)	1.4 (58.4)[e]
30 mg	14	387 (99.4)	1010 (92.3)	1369 (59.3)[f]	1.9 (0.9–3.1)	2.9 (38.7)[f]

AUC_{0-t}, area under the concentration-time curve from time 0 to time t; *$AUC_{0-\infty}$*, area under the concentration-time curve from time 0 to time infinity; *C_{max}*, maximum observed plasma concentration; *$t_{1/2}$*, half-life; *t_{max}*, time to maximum observed plasma concentration.
[a]Geometric mean (%CV).
[b]Median (range).
[c]n = 12.
[d]n = 9.
[e]n = 8.
[f]n = 11.
