# Supplementary material for: A randomized, 29-day, dose-ranging, efficacy and safety study of daprodustat, administered three times weekly in patients with anemia on hemodialysis
Source: BMC Nephrol. 2019 Oct 16;20:372. doi: 10.1186/s12882-019-1547-z (PMC6796426; doi:10.1186/s12882-019-1547-z)
Supplement: Supplementary file 2 — Participating research centers and IEC/IRB committees. (PDF 94 kb) [file 12882_2019_1547_MOESM2_ESM.pdf]

Additional File 2 Participating research centers and IEC/IRB* committees

	Description of Research Facility, Hospital/Institution and Address	Name of IEC/IRB Committee and Address
**Canada**		
	Centre Intégré de Santé et de Services Sociaux de la Montérégie-Centre (CISSSMC), Greenfield Park, Québec, Canada	Comite d'ethique de la recherche de l'Hôpital Charles-Le Moyne/CISSS de la Montérégie-Centre, E-302, 3120 Boulevard Taschereau, Greenfield Park, Québec, J4V 2Hl, Canada
	Humber River Regional Hospital, Nephrology Clinical Trials Office, Toronto, Ontario, Canada	Humber River Hospital Research Ethics Board C/O Irene Melissa, 1235 Wilson Avenue, Room 3C3014, Toronto, Ontario, M3M 0B2, Canada
	Sheldon M. Chumir Health Center/Alberta Health Services Calgary, Alberta, Canada	Conjoint Health Research Ethics Board (CHREB), #300, 3rd Floor MacKimmie Library Tower, 2500 University Drive NW, Calgary, Alberta, T2N 1N4, Canada
	The Ottawa Hospital, Ottawa, Ontario, Canada	Ottawa Health Science Network Ethics Board 725 Parkdale Avenue, Ottawa, Ontario, KIY 4E9, Canada
**Germany**	**All German requests were approved through the same IEC/IRB** Ethik-Kommission der Sächsischen Landesärztekammer, Schützenhöhe 16, Dresden, 01099, Germany	
	Robert Bosch Krankenhaus, Abt.f.Allg. Inn. Med u. Nephrologie, Stuttgart, Baden-Wuerttemberg, Germany	
	Universitaetsklinikum Schleswig-Holstein, Klin f. Inn. Medizin, Klin f. Nieren- und Hochdruckkrankheiten, Kiel, Schleswig-Holstein, Germany	
	Dialyse Centrum - Darmstadt, Nephrologische Gemeinschaftspraxis, Darmstadt, Germany	
	Nephrologisches Zentrum Villingen-Schwenningen, Albert-Schweitzer-Villingen-Schwenningen, Baden-Wuerttemberg, Germany	
**Russia**	**In addition to local offices, all IRB and Ethics requests in Russia were approved through:** The Russian Federation Ministry of Healthcare, Department of State Regulation of Circulation of Medicines, Ethics Council, 3, Rakhmanovskypereulok, Moscow, 127994, Russia	
	Budgetary Institution of Healthcare of Omsk Region, City Clinical Hospital #1 named after A.N. Kabanov, Omsk, Russia	Ethics Committee at Budgetary Institution of Healthcare of Omsk Region, City Clinical Hospital # 1 named after Kabanov, Ulitsa Pereleta, 7, Omsk, 644112, Russia
	LLC Fresenius Medical Care Kuban, Krasnodar, Russia	Ethics Committee at Fresenius Medical Care Kuban LLC, 22, ulitsa Cherkasskaya, Krasnodar, 350029, Russia
	Nephroline-Novosibirsk LLC, Novosibirsk, Russia	Ethics Committee at Nephroline-Novosibirsk LLC, 143/1, ulitsa Nemirovicha-Danchenko, Novosibirsk, 630025, Russia
	Fresenius NephroCare LLC, ulitsa, Penza, Russia	Ethics Committee at Fresenius NephroCare LLC, 35, ulitsa Valovaya, Moscow, 115054, Russia
	Dialysis Center LLC, Peritoneal Dialysis Department, Podolsk, Russia	Ethics Committee at Dialysis Center LLC, 35, ulitsa Valoyava, Moscow, 115054, Russia
	Fresenius (LLC Dialysis Center), Mytischi, Russia	Ethics Committee at Dialysis Center LLC, 35, ulitsa Valoyava, Moscow, 115054, Russia
	Saint Petersburg State Budgetary Institution of Healthcare, City Clinical Hospital # 31, Saint-Petersburg, Russia	Ethics Committee at Saint Petersburg State Budgetary Institution of Healthcare, City Clinical Hospital # 31, Prospect Dinamo 3, Saint-Petersburg, 197110, Russia

Additional File 2 Participating research centers and IEC/IRB* committees

	State Budgetary Institution of Healthcare of Yaroslavl Region, Yaroslavl Regional Clinical Hospital, Cardiovascular Department, Yaroslavl, Russia	Ethics Committee at State Budgetary Institution of Healthcare of Yaroslavl Region, Regional Clinical Hospital, 7, ulitsa Yakovlevskaya, Yaroslavl, 150062, Russia
	Dialysis Center Saint Petersburg LLC, Saint Petersburg, Russia	Local Ethics Committee at Dialysis Center Saint Petersburg LLC, 1, litera A, the Northern Avenue, Saint Petersburg, 194354, Russia
**Spain**	**All IRB and Ethics requests in Spain were approved through the same IEC/IRB:** Comite Etico de Investigación Clinica del Hospital Universitario 12 de Octubre, Avenida de Cordoba s/n, Madrid, Madrid, 28041, Spain	
	Hospital Universitario Infanta Sofia, Department of Nephrology, San Sebastian de los Reyes, Spain	
	Centro de Diálisis de Alcobendas, Centro Diálisis Alcobendas. Fresenius Medical Care Madrid S.A., Diálisis, Madrid, Spain	
	Hospital Universitari Germans Trias I Pujol, Badalona, Barcelona, Spain	
	Hospital General Universitario de Guadalajara, Servicio de Nefrologia, Guadalajara, Spain	
	Complejo Hospitalario Torrecardenas, Hospital de Torrecárdenas, Almería, Spain	
	Complejo Hospitalario Universitario de Santiago, Servicio de Farmacia, Santiago de Compostela, A Coruna, Spain	
	Hospital de Manises, Servicio de Nefrologia, Avenida Generalitat Manises (Valencia), Spain	
	Fundacion Jimenez Diaz 1, Servicio de Nefrologia, Madrid, Spain	
	Hospital Regional Universitario de Malaga, Pulmonology/Pneumonology Department, Malaga, Spain	
	Hospital Universitario Nuestra Senora de La Candelaria, Servicio de Nefrología, Santa Cruz De Tenerife, Spain	
	Hospital Universitario Marques de Valdecilla, Avenida. Marques de Valdecilla, Cantabria, Spain	
	Corporacio Sanitaria Parc Tauli, Servicio de Nefrologia, Edificio Albada, Barcelona, Spain	
	Centro de Diálisis de Granollers, Fresenius Medical Care Services Catalunya S.L, Diálisis, Barcelona, Spain	
	Centro de Diálisis de Terrassa, Fresinius Medical Care Services Catalunya S.L, Diálisis, Barcelona, Spain	
	Hospital del Mar, Servicio De Nephrologia, Barcelona, Spain	
	Hospital Universitario 12 de Octubre, Servicio de Nefrologia., Madrid, Spain	
	Centro de Dialisis Leon. Fresenius Medical Care Services Castilla y Leon, S.L., Dialisis, Leon, Spain	

Additional File 2 Participating research centers and IEC/IRB* committees

	Centro de Dialisis Valladolid,<br>Fresenius Medical Care Services<br>Castilla y Leon, S.L., Dialisis, Valladolid, Spain
	Hospital Universitari Arnau de Vilanova,<br>Servicio de nefrología,<br>Lleida, Spain
	Hospital Universitario de Bellvitge,<br>Feixa Llarga s/n, L'Hospitalet de Llobregat,<br>Barcelona, Spain
	Hospital General Universiatrio de<br>Ciudad Real, Nefrología, Unidad de Dialisis,<br>C/ del Obispo Rafael Torija, Ciudad Real, Spain
**United States**	**All IRB and Ethics requests in the United States were approved through the same IEC/IRB:**<br>Quorum Review Institutional Review Board, Suite 800, 1501 Fourth Avenue,<br>Seattle, Washington, 98101, United States
	Michigan Kidney Consultants, PC<br>Pontiac, Michigan, United States
	Paragon Health // PC DBA<br>Nephrology Center Kalamazoo, Michigan, United States
	Clinical Research Consultants, LLC<br>Kansas City, Missouri, United States
	Valley Renal Medical Group<br>Clinical Research Center<br>Northridge, California, United States
	North America Research Institute<br>San Dimas, California, United States
	Seacoast Kidney & Hypertension Specialists<br>Portsmouth, New Hampshire, United States
	San Diego Institute of Medical Research<br>Escondido, California, United States
	Nephrology & Hypertension Associates<br>Waters Place, Bronx, New York, United States
	A. Kaldun Nossuli MD/Research<br>Greenbelt, Maryland, United States
	Saint Clair Nephrology Research<br>Roseville, Michigan, United States
	Kansas Nephrology Research Institute LLC<br>Wichita, Kansas, United States
	Downey Dialysis Center<br>Downey, California, United States
	Pines Clinical Research Inc.<br>Hollywood, Florida, United States

*Institutional Review Board reference numbers are not available.
